# Using Economic Evaluation to Hasten Health Equity

**DOI:** 10.1089/heq.2021.0010

**Published:** 2021-09-15

**Authors:** Maria Isabel Roldós, Nancy Breen

**Affiliations:** ^1^ City University of New York (CUNY) Institute for Health Equity, Bronx, New York, USA.; ^2^ Department of Health Services Administration, School of Health Sciences, Human Services and Nursing (HS2N), Lehman College–CUNY, Bronx, New York, USA.; ^3^ Office of Science Policy, Planning, Evaluation, and Reporting (OSPPER), National Institute on Minority Health and Health Disparities, National Institutes of Health, Bethesda, Maryland, USA.

**Keywords:** economic evaluations, health equity polices, HRQL, WTP, CBAs

## Abstract

Achieving health equity has proven elusive for two reasons. First, most research has focused on changing the behavior of individuals; however, policies that address socioeconomic factors or change the context to facilitate healthy decisions tend to be more effective. Second, health disparity science and evidence are not consistently used to guide policy makers, even those seeking health equity. In this perspective, we discuss economic evaluation tools that researchers can use to assist decision-makers in conducting research or evaluating policy: self-reported health-related quality of life surveys and cost–benefit analysis evaluations informed with willingness to pay research and analyses.

## Introduction

Describing health disparity does not necessarily translate into health equity programs or policies. If, as is widely believed, health disparities are the outcomes of multiple causes operating at multiple levels in multiple domains that play out over a long period of time,^1^ then policies that change the context in which people make decisions will have a greater opportunity to improve population health.^2^ For studies to be useful to policy makers, researchers need to delineate the characteristics of the population at high risk; understand fundamental causes of those disparities; design interventions to address the causes; and quantify health improvements.^3,4^ In addition, researchers need to build strong partnerships with communities and policy makers to identify priorities. The challenge is that communities are faced with competing priorities with limited budgets, and decision-makers need results from tools that identify the most pressing priorities of communities as well as priorities with the greatest potential of demonstrating return on investment.

This perspective proposes tools to promote evidence-based policy making by promoting the use of self-reported health-related quality of life (HRQoL) surveys (to characterize populations experiencing health disparities and measure health outcomes before and after interventions) and cost–benefit analyses (CBAs) informed by willingness to pay (WTP) analyses of samples from diverse backgrounds that have the potential to capture the economic benefit of intangible health improvements. We discuss these methods and conclude with recommendations to help bridge research and policy.

## HRQoL Surveys

Essential to successful policy is characterization of a population's health profile and those experiencing health disparities. Health disparity populations are those that experience disparity in the health outcome, such as an increased overall rate of disease incidence, prevalence, morbidity, mortality, or lower survival compared with the general population.^1^ The National Institutes of Health (NIH) designated US health disparity populations as all the US Office of Management and Budget (OMB)-defined racial and ethnic minorities; socioeconomically disadvantaged populations; underserved rural populations; and sexual and gender minorities to promote research and improve the characterization of the etiology of health inequality.^5^ Because health disparity populations experience more risk factors and comorbidities than the general population, health profile tools are needed to demonstrate the assets and deficits of the population at the baseline of the project as well as to compare with other populations and identify the causes of disparities.

A reliable tool for quantifying health profiles is the self-reported HRQoL survey. HRQoL surveys measure physical, occupational, and psychological functions, social interaction, and somatic sensations.^6^ Established standardized instruments are EQ-5D and SF-36.^7,8^ EQ-5D measures five scales: mobility, self-care, usual activities, pain/discomfort, and anxiety/depression.^9^ SF-36/12 measures eight scales that yield two composite summary scales: physical health (bodily pain and general health) and mental health (vitality; social functioning; and emotional and overall mental health). For example, research using SF-36 found that Asian Americans conceptualize mental and physical health differently from Western populations to suggest that their cultural systems shape their health perceptions, behaviors, and coping strategies.^7^ This study illustrates a specific population's HRQoL and presents the opportunity to compare it with other racial and ethnic minorities as well as with their White counterparts. The field of health disparities research (HDR) calls for the comparison of health outcomes and health burdens within and between populations to better understand the causes and to later design interventions to address the causes.^10^

In addition, an essential, but often overlooked, point is that HRQoL data should be collected in ways that make them reliable to construct composite scores representative across populations experiencing health disparities.^11^ HRQoL data can be used to construct health outcome measures such as quality-adjusted life years (QALYs). QALYs combine the length and quality of life into a single index to allow comparison of competing alternatives with different health outcomes.^12^ QALYs are calculated by multiplying the duration of time spent in a health state by the HRQoL weight (i.e., utility score) associated with that health state. The QALY approach allows comparison of health outcomes between populations and is widely used in cost-effectiveness studies in health service research. In this perspective, we argue that HRQoL data from diverse populations can be used as a stand-alone health outcome metric as well as to develop representative population profiles and indices, estimate utility scores, and construct improved composite measures.

## Estimating the Benefits of Reducing Health Disparities Using WTP and CBAs

An article published in *Lancet* in 2002 reported that despite the importance of economic assessment of health care interventions in multiple countries, the UK leader, the National Institute of Clinical Excellence (NICE), accepted only evidence from cost-effectiveness assessments (CEAs) and cost–utility analyses.^13^ The authors argued that cost–benefit analysis (CBA) should be added because CBA is designed for comparison among different health care interventions and different allocations of public spending. Subsequently, NICE expanded its third edition of *Methods for the development of NICE public health guidance*, published in 2012, to place more emphasis on cost–benefit analyses when assessing public health interventions.^14^

CBA examines whether a policy's benefits are worth the investment. It can be used to evaluate economic trade-offs among policy alternatives because both costs and benefits are measured in monetary terms.^15^ The key advantage of CBA for health disparities research is that it can evaluate the economic trade-offs between policy alternatives that address social determinants. The challenge in any CBA is to estimate the economic value of health improvements or of potential lives saved.^15^ This is the purpose of WTP studies. The World Health Organization (WHO) recommends using CBA and WTP studies to monetize the desired outcome.^16^ In social CBA, social benefits are measured based on individuals' preferences for the desired outcome. Other economic evaluation tools, such as CEA, use health improvement metrics as outcomes in evaluations, while CBAs use money as the outcome metric.

For outcomes without a price, WTP studies can be used to quantify the costs averted, especially when the benefits represent an intangible health improvement.^16^ WTP studies contribute to measurement of the benefit side of a CBA. Traditional benefit metrics include improvements in labor productivity and reduction of medical expenditures, direct and indirect. WTP has the greatest potential to measure intangible benefits often associated with mental and emotional health, as well as philanthropic values. WTP studies can assess public goods not traded in the marketplace, such as clean water or a society free of health disparities. WTP tools pose questions related to the individual's willingness to sacrifice financial resources to avoid risk, such as premature death, by presenting a hypothetical scenario. For example, a study about child maltreatment posed the following scenario: “If you were presented with an intervention or program with evidence of effectiveness that would reduce child mortality related to violence, how much would you pay to have that program in your community?”^17^ Thus, the logic of the method is that respondents are asked to think about a public good as if it could be purchased in a marketplace.^18^ Then, the amount of money an individual would pay to avoid premature death is used to estimate the value of a statistical life (VSL).^19^ VSL summarizes the WTP estimate for mortality risk reduction and is used to calculate benefits in a CBA. Traditional methods underestimate benefits for those from disadvantaged backgrounds due to which these rely on the human capital approach. To the point of this perspective, the estimate of the WTP study will allow for more benefits to be included and demonstrate that the benefits outweigh the costs. This is particularly needed among underserved communities that cannot demonstrate the economic benefit of prevention programs in their communities and attract public monies and political interest.

Furthermore, VSL describes an individual's marginal rate of substitution between money and mortality risk in a defined time period (e.g., the current year).^20^ Central to WTP measurement and VSL estimation is that individuals use preferences and past experiences to value reduction in mortality risk.^21^ Research has shown that the VSL will vary substantially depending on age, income, and other sociodemographic variables of respondents^22^; preferences also will vary by source of risk^23^ and type of health outcome.^24^ VSL estimates are commonly derived from wage differentials for on-the-job risk exposure,^25^ but wage differentials are not suitable for population-based interventions. CBA is used by the US federal government to evaluate proposed regulations. The OMB provides recommendations for estimating the VSL; however, it is worth noting that VSL estimates differ widely across US federal agencies, ranging from $1 million to $10 million per statistical life.^26,27^ Therefore, we recommend including larger samples of populations with disparities when conducting WTP studies to allow for variation in age and other risk factors within populations with health disparities.

VSL estimates from the WTP values are used to calculate the portion of benefits in the CBA evaluation. Benefits are computed as the expected deaths avoided by the policy change times the average WTP value.^19^ VSL estimates refer to the aggregate measurement of WTP. They do not suggest that any individual's life can be expressed in monetary terms. Their sole purpose is to estimate the monetary benefit of a regulatory action.

An important advantage of CBA is that it is straightforward to evaluate multiple impacts. Potential impacts of social determinants of health (SDOH) policy interventions on social welfare include improvements in life expectancy, HRQoL, cognitive development, behavior, social competence, educational attainment, and earnings and reduced delinquency and crime. Economic methods have been developed to estimate WTP for many of these outcomes. The WHO report concludes that CBA provides the most comprehensive approach to evaluate SDOH interventions.^16^

## Recommendations

The tools described in this perspective are rarely used in health disparities research and we believe they should be included when advocating for policy implementation of interventions shown to reduce health disparities. Some interventions that work well may be too expensive. If so, can the intervention be refined so that it still reduces health disparities, but is less expensive to implement? Continual monitoring is needed to ensure that the policy is working as anticipated and, if not, to modify it in a timely manner. In the following sections, we propose specific recommendations for health disparities researchers and for those promoting policies to reduce them.

Box 1 proposes three specific recommendations for researchers. First is to administer HRQoL surveys to populations that experience disparities so as to obtain representative population profiles and outcome indices. Collecting data that reflect their preferences and perceptions will make the data more representative. Researchers have the opportunity to use HRQoL as a stand-alone metric to describe population health. In fact, the Centers for Disease Control and Prevention (CDC) panel on CEAs has recommended the use of alternative health outcome measures for public health studies and evaluations and reevaluated the general use of QALYs as the sole health outcome measure for CEAs.^28^ HRQoL data from diverse backgrounds will also permit the comparison of HRQoL between specific populations and their counterparts. These comparisons will then allow for identification of the causes of disparities. HDR calls for identification of causes with particular interest in those related to social determinants of health.^29^ In addition, more diversity in HRQoL data has the potential to address the theoretical and practical problems with the QALY. Second, studies need to measure WTP and VSL—for use in CBA evaluation—in ways that are valid and reliable across populations that experience health disparities. This will facilitate comparison across studies, regardless of population profile or outcome, and identify causes. Best methodological practices to estimate WTP are published elsewhere.^30^ Third is to present information derived from HRQoL, WTP, and VSL to communities experiencing health disparities to collaboratively develop interventions that thoroughly and cost-effectively address SDOH and to establish benchmarks for evaluation. Incorporating the needs and preferences of representative individuals in a community is critical to building sustainable interventions.^31^ HRQoL data should be collected and reported initially and periodically so that the community can perceive the initial health disparities, need for intervention, and what occurs after the intervention is implemented.

**Box 1.** Recommendations to Hasten the Science of Health Disparities Research

(1) Collect HRQoL data from populations experiencing health disparities using preference-based measures or self-report instruments to develop representative population profiles and indices. (2) Conduct WTP studies and estimate the VSL of the population under study. (3) Present results from steps 1 and 2 to the study population and collaborate on interventions that address SDOH. Work with the community and policy makers to establish benchmarks for evaluating success.

HRQoL, health-related quality of life; SDOH, social determinants of health; VSL, value of a statistical life; WTP, willingness to pay.

Policy makers need to be included strategically throughout the process. Box 2 describes recommendations to support decision-makers seeking to develop health equity policies. Comparing alternative policies promotes transparency and justifies priorities. HRQoL, WTP, and CBAs illustrate policy feasibility and return on investment. First, HRQoL data can be used to characterize a population's well-being, anticipated health improvements resulting from policies, and how progress will be monitored. Policies that seek to improve health, prevent death, and reduce disparities take time. Researchers can use HRQoL data to demonstrate how and when outcomes will be evaluated and when results are anticipated. This information allows populations and policy makers to adjust expectations and ensure that adequate funding will be provided long enough for the project to actually reduce health disparities. Intermediate outcomes should be designed to show progress and that reducing health disparities is feasible. Second is to present the costs and benefits of policies using money as the unit of analysis. The societal perspective of CBAs allows policy makers to learn how the costs and benefits of addressing SDOH directly compare with other policies and expenditures designed to reduce health disparities. As an estimate of the benefit of eliminating health disparities, the United States would have saved about $230 billion in direct medical care expenditures and more than $1 trillion in indirect costs associated with illness and premature death for the years 2003–2006.^32^ Demonstrating positive net costs for the economic burden of health disparities has the potential to attract sustained funding. For example, in 2020, the US Congress Tri-Caucus—consisting of the Congressional Asian Pacific American Caucus (CAPAC), the Congressional Black Caucus (CBC), and the Congressional Hispanic Caucus (CHC)—introduced a comprehensive and strategic legislative roadmap that aims to eliminate racial and ethnic health disparities, entitled the Health Equity and Accountability Act (HEAA). The HEAA is designed to avoid future health care costs associated with inequality in the US health care system.^33^

**Box 2.** Using HRQoL Health Profiles, WTP, and VSL Data to Drive Policy Making

(1) Use HRQoL data to present baseline measures, community preferences, and anticipated results so that policy makers can envision outcomes and the time required to achieve them. (2) Use CBAs to estimate costs so that policy makers can compare the costs and benefits with other policies.

CBAs, cost–benefit analyses.

## Limitations

Broad limitations need to be considered, without undermining the usefulness of the methods discussed in this perspective. First, there is no agreed-upon VSL and this is an area that needs more research. VSL varies depending on the characteristics, including age, of the life that is being valued. In fact, older adults may be valued less than the younger populations due to the formula used for calculation.^34^ Therefore, we recognize that the whole idea of valuing life is confusing and controversial to the general public.^35^ Second, within the field of economic evaluation, there is debate relating to whether all QALYs are the same, with questions about who should value health states.^36^ Further research on the QALY approach is needed to address challenges concerning equity-weighted utility maximization and testing of the validity of underlying assumptions. Finally, a specific limitation of HRQoL is that it is collected through self-report instruments. As with many self-report measures, individuals may not assess themselves accurately and, for example, may respond with more socially acceptable answers than their actual health status, skewing evaluations.

These methodological challenges need to be addressed by bringing together experts from different disciplines, such as economics and health disparities, to propose ways to overcome these challenges and to make metrics more reliable, valid, and unbiased for populations with health disparities.

## Conclusions

HRQoL data have been associated with CEAs and WTP data with CBAs. The current use of these tools is mainly in health economics or outcome research, and we argue for them to advance the science of HDR. Our main recommendation is that HDR would be strengthened if CBAs were used to examine policies addressing SDOH. Increasingly, health disparity causes are upstream and require policy interventions. Advocacy for successful interventions to become policy requires cost estimates because, invariably, policy recommendations to address SDOH interventions proven to reduce health disparities are competing with other budget items. The tools and methods described in this perspective have the potential to strengthen HDR by improving our ability to provide reliable, valid, and unbiased metrics and analyses, including CBAs and allow comparisons within and between populations and identify causes.

Interdisciplinary collaboration among researchers is needed to do this. CBA is important because it summarizes benefits and costs in the same unit, money. The Pew Charitable Trusts and the MacArthur Foundation collaborated on an initiative to promote evidence-based transparent policy making. Their report, *Results First*, found that the number of states assessing the costs and benefits of programs and policy options increased by 48% between 2008 and 2011. Twenty-nine states reported using cost–benefit studies to inform policy or budget decisions. Since 2011, 16 states and four California counties have partnered with the Results First Initiative to apply a customized, innovative cost–benefit approach to policy and budget decision-making.^37^ *Results First* invites researchers and policy makers to use CBAs more widely and focus on demonstrating the economic benefits of policies that promote health equity. We agree that CBA evidence is an important tool in economic assessment and believe it should be added to health disparities research toolkits in the United States.

## Abbreviations Used

CAPAC: Congressional Asian Pacific American Caucus
CBA: cost–benefit analysis
CBC: Congressional Black Caucus
CDC: Centers for Disease Control and Prevention
CEAs: cost-effectiveness assessments
CHC: Congressional Hispanic Caucus
CUA: cost–utility analyses
HEAA: Health Equity and Accountability Act
HDR: health disparities research
HRQoL: health-related quality of life
NICE: National Institute of Clinical Excellence
NIH: National Institutes of Health
NIMHD: National Institute on Minority Health and Health Disparities
OMB: Office of Management and Budget
OSPPER: Office of Science Policy, Planning, Evaluation, and Reporting
QALYs: quality-adjusted life years
SDOH: social determinants of health
VSL: value of a statistical life
WHO: World Health Organization
WTP: willingness to pay
